# The Importance of Functionally Characterizing Calcium-Sensing Receptor Variants in Individuals With Hypercalcemia

**DOI:** 10.1210/jendso/bvac052

**Published:** 2022-04-04

**Authors:** Caroline M Gorvin

**Affiliations:** 1 Centre of Membrane Proteins and Receptors (COMPARE), University of Birmingham, Birmingham, UK; 2 Institute of Metabolism and Systems Research (IMSR) and Centre for Diabetes, Endocrinology and Metabolism (CEDAM), University of Birmingham, Birmingham, UK

**Keywords:** familial hypocalciuric hypercalcemia, genetic variants, hypocalcemia, pathogenicity prediction

Germline variants in the calcium-sensing receptor (CaSR), a G protein–coupled receptor that regulates parathyroid hormone (PTH) secretion, are associated with disorders of calcium homeostasis. Inactivating mutations most often cause familial hypocalciuric hypercalcemia (FHH), a lifelong condition characterized by elevated serum calcium values, high-to-normal PTH concentrations and low renal calcium excretion. Individuals with FHH are frequently asymptomatic (~71%) [1] and do not require treatment; however, the biochemical features of FHH have considerable overlap with typical primary hyperparathyroidism (PHPT), and an accurate diagnosis of the 2 conditions is important to prevent unnecessary parathyroidectomy. Although urinary calcium/creatinine clearance ratios (UCCRs) can differentiate between the 2 conditions in many cases, some patients with PHPT (eg, those with vitamin D deficiency or renal insufficiency) can also present with low UCCR [2, 3]. Therefore, genetic analysis is still the gold standard in differentiating between FHH and PHPT. However, with the reduced costs and relative ease in performing next-generation sequencing over the past decade, vast numbers of genetic variants have been detected within human populations, and it is increasingly difficult for diagnostic teams to determine causality of individual variants, particularly in cases where cosegregation analysis cannot be performed (eg, lack of samples from family members).

Alongside the rapid increase in sequencing data, a number of online tools have become available that aid in the interpretation of sequence variants that use simplistic terms such as pathogenic vs benign. Each tool differs in their precise algorithms; however, most base their predictions on either evolutionary conservation, biochemical consequences of amino acid substitutions, or a combination of these [4]. As these tools are inherently designed for ease of use, and are increasingly incorporated within genome browsers such as Ensembl or NCBI, researchers can become over-reliant on them and neglect to question their accuracy. Moreover, the precision of these tools is particularly problematic for disorders with milder phenotypes, such as FHH that can go undiagnosed in the general population, as demonstrated in a cohort of 51 289 individuals in a single healthcare population in which 12 functionally inactivating CaSR variants were identified [5]. Furthermore, some CaSR variants have been identified incidentally in patients with FHH, but subsequently shown to have no pathogenic effect, or shown to be functionally activating when later identified in hypocalcemic individuals [6]. A visit to the genome aggregation database (GnomAD, https://gnomad.broadinstitute.org/) identifies almost 400 missense coding variants (accession March 15, 2022), most of which are rare and have unknown effects on CaSR activity. Therefore, characterization of CaSR variants identified in individuals with FHH is important for correct diagnoses.

A recent report by Mullin et al [7] highlighted the problem in correlating pathogenicity predictions with the functional effect of CaSR variants. They identified 3 previously unreported CaSR variants in patients with FHH, which upon bioinformatic analysis using 8 different tools had inconsistent findings, resulting in a conclusion that these were “variants of unknown significance,” a scenario that will be common to many geneticists and clinicians. Functional analysis of the variants showed impaired protein expression and reduced activity, consistent with the variants causing the observed FHH phenotype [7]. Importantly, when the group expanded their analyses to examine the utility of prediction algorithms to 23 functionally characterized disease-causing and 4 benign (non-disease) CaSR variants, many of these tools were shown to be ineffective in accurately predicting pathogenicity. For example, CADD-Phred predicted ~70% of known functionally inactivating variants as benign [7]. In contrast, Polyphen-2 correctly predicted all 23 FHH variants as possibly or probably damaging. However, it also predicted 3 of the 4 CaSR variants that are not associated with changes in functional activity as pathogenic, suggesting that Polyphen-2 overestimates the deleteriousness of variants. By comparing pathogenicity predictions to elevated EC_50_ values of variants from a single study, and thus avoiding inherent variation due to assay differences, the authors concluded that there is no appreciable correlation between functional effects and predicted pathogenicity [7]. This is consistent with the American College of Medical Genetics and Genomics findings that most algorithms are 65% to 80% accurate when estimating known disease variants [4]. Thus, for CaSR variants identified in individuals with hyper/hypocalcemia, pathogenicity prediction should never be used in isolation to assign causation, and should be combined with knowledge of disease-causing variants (eg, previously characterized missense variants at the same position/mutational hotspots); in silico modeling, which has become easier with the recent publication of several near full-length CaSR cryo-electron microscopy structures [8]; or preferably functional analysis examining protein expression and CaSR activity [4]. Following these guidelines should yield accurate patient diagnoses and improve patient care.

## Abbreviations

CaSR: calcium-sensing receptor
FHH: familial hypocalciuric hypercalcemia
PTH: parathyroid hormone
PHPT: primary hyperparathyroidism
UCCR: urinary calcium/creatinine clearance ratio
